# Risk factors for early mortality of lung cancer patients in France: A nationwide analysis

**DOI:** 10.1002/cam4.4821

**Published:** 2022-05-14

**Authors:** Hélène Goussault, Sébastien Gendarme, Jean‐Baptiste Assié, Camille Jung, Salomé Epaud, Christelle Algans, Noémie Salaun‐Penquer, Mathilde Rousseau, Andrea Lazatti, Christos Chouaïd

**Affiliations:** ^1^ Département de Pneumologie CHI de Créteil Créteil France; ^2^ Centre de Recherche Clinique CHI de Créteil Créteil France; ^3^ Kaduceo Toulouse France; ^4^ Department d'Information médicale CHI de Créteil Créteil France; ^5^ Département de Chirurgie Digestive CHI de Créteil Créteil France; ^6^ UPEC, Inserm U955, IMRB Créteil France

**Keywords:** early mortality, epidemiology, lung cancer, mortality, prognosis

## Abstract

**Background:**

Despite therapeutic advances, lung cancer remains the first cause of death from cancer. The main objective of this study was to identify risk factors associated with death within 3‐months of the first hospitalization for lung cancer in France.

**Methods:**

This analysis included patients with a first hospitalization for lung cancer (between January 1, 2016 and December 31, 2018) according to diagnosis‐related groups entered into the French national medical‐administrative database. Clinical and socioeconomic parameters and characteristics of that first hospitalization were analyzed. A model predictive of early mortality was developed based on those variables.

**Results:**

The 144,087 included patients were 67% men; median age of 68 [interquartile range 60–76] years; 47% had metastatic disease at diagnosis; and 34% and 23%, respectively, had received systemic treatment or undergone curative surgery. The 3‐month mortality was 19%, and significantly higher for those ≥70 versus <70 years old (OR 1.33, 1.22–1.45), men versus. women (OR 1.50, 1.44–1.55), those with metastatic disease at diagnosis (OR, 3.30, 3.18–3.43), first hospitalization via the emergency room (OR 1.65 1.59–1.71) and first hospitalization lasting >30 days (OR, 1.58 1.49–1.68). In contrast, no socioeconomic characteristic was associated with early mortality.

**Conclusion:**

Almost 1 in 5 patients diagnosed with lung cancer in France died within 3 months post‐diagnosis. Improving survival requires diagnosis at an earlier stage and better organization of diagnosis and specific care pathways.

## Graphical abstract

In France, almost 1 in 5 patients diagnosed with lung cancer died within 3 months following first hospitalization. Early mortality is higher for patients 70 years, for men, for those with metastatic disease at diagnosis, for patients hospitalized via the emergency room and with first hospitalization lasting >30 days.

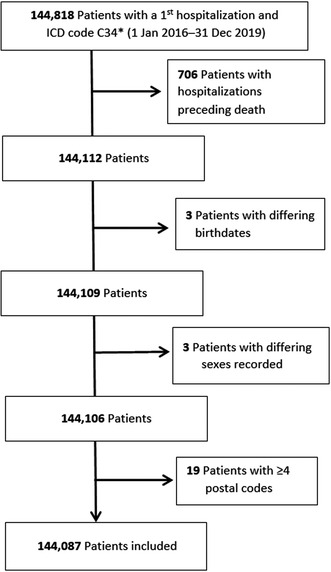

## 1 INTRODUCTION

Lung cancer today is the primary cause of cancer‐attributed deaths worldwide.^1^ Even if, due to recent therapeutic advances, including targeted therapy and immunotherapy, mortality trends to decreased quickly since the last decade (by 6.3% annually for men and 5.9% annually for women in NSCLC from 2014 through 2016 in the US,^2^ prognosis remains pejorative. That finding suggests individual poor‐prognosis factors, independent of therapeutic management, are at play. Survival is significantly associated with the disease stage at diagnosis.^3^ In France, 5‐year survival of all stages combined is estimated at 14%, but almost half of the patients had metastatic disease at diagnosis and their 5‐year survival is only 3.8%.^4^ In addition to the stage, tumor histology, oncogenic addiction, sex, and smoking status are significantly linked to median survival.^5^ Disparities in access to care and socioeconomic factors also underlie survival differences, probably linked to delays in diagnosis and management.^6^, ^7^

However, data are fragmented on the importance of factors linked to the early mortality of patients diagnosed with lung cancer. Indeed, no consensus has been reached on the definition of early mortality. For lung cancer patients who underwent surgery, the cut‐off was often 1 month^8^, ^9^, ^10^; for locally advanced stages treated with radio‐chemotherapy, some studies retained 6 months or 1 year,^11^, ^12^, ^13^ but most of those studies chose 3‐months after histological diagnosis or the onset of therapeutic management.^14^, ^15^, ^16^, ^17^, ^18^, ^19^

The principal objective of this study was to identify, using the French national medical‐administrative database, the early mortality rate and the risk factors associated with death within 3 months of the first hospitalization for lung cancer, in the general population of non‐selected lung cancer patients, and subgroups of patients who had received at least one systemic treatment or had undergone curative surgery. The secondary aim was to develop a model predictive of early mortality for lung cancer patients.

## 2 METHODS

This retrospective, longitudinal study was based on Diagnosis‐Related Groups (DRG) from the French national medical‐administrative database, which includes all hospitalizations in public and private facilities in France.^20^ The reasons for hospitalization are coded by diagnosis according to the International Classification of Diseases, Tenth Revision, Clinical Modification (ICD‐10‐CM). Available information includes age, sex, residential postal code, comorbidities, admission from and discharges to details and the duration of hospitalizations and treatments received, particularly systemic anti‐cancer treatments and surgical interventions. A single identifier allows the follow‐up of patients throughout their management. A patient's in‐hospital death is documented but the cause is not specified.

The analysis included all adult patients first hospitalized for lung cancer, including one‐day hospitalization, between 1 January 2016 and 31 December 2018. These patients were identified by the ICD‐10 code C34* (malignant neoplasm of bronchus and lung). To include only incident cases, the patients who had been hospitalized for lung cancer during the 4 preceding years were excluded. The codes corresponding to cancer stage, curative surgery, and systemic treatment were only sought during the 3 months following the first hospitalization to avoid bias related to the natural evolution of the disease.

The 2020 data from the Institut National de la Statistique et des Études Économiques (INSEE),^21^, ^22^ based on residence postal code, classed each patient's home as being urban or rural, with 4 population density classes.^23^ Also, accessibility to care in the patient's residential district was assessed using an indicator^23^ that distributed the population into 4 classes. Finally, based on data available for 2014, patients' districts of residence were classified into 4 classes, according to the Social Deprivation Index (SDI), determined from the district's unemployment rate, median household revenue, the percentage of high school diplomas in the adult population and the percentage of blue‐collar workers in the active population^24^

From the Fichier National des Établissements Sanitaires et Sociaux (FINESS), healthcare facilities managing the patients were classified as university hospitals, general hospitals, dedicated cancer centers, or other public or private facilities.^25^

The main judgment criterion was early death—from all causes combined—within 3 months of the first admission for lung cancer in the general lung cancer population. This threshold was chosen to reflect the management of this cohort, where the majority of patients are a priori metastatic.

Quantitative data, expressed as median (interquartile range [IQR]), were compared with Student's t‐tests between patients dying within 3 months of first hospitalization and those surviving beyond that time. Qualitative data, expressed as numbers (%), were compared between those 2 groups with chi^2^ tests. The significant threshold was set at *p* ≤ 0.05.

Factors significantly associated with early mortality in the univariate analyses (achieving *p* ≤ 0.05) were included in the multivariate analysis. Only comorbidities present in >10% of the total population were integrated into the logistic regression multivariate analysis. The results are expressed as odds ratio (OR) [95% confidence interval (CI)].

No correction was made for missing, outlier, or aberrant values. The predictive model relies on the variables significantly associated with early deaths. The patients with missing data among the selected variables were excluded from the model. Different classification algorithms were tested: logistic regression, decision trees, and support vector machines (SVMs, i.e., supervised learning models). The choice of model explicative variables was adjusted by backward‐stepwise elimination from the selection algorithm. Oversampling was used to limit model bias toward the population of patients not dying early. To achieve high sensitivity, the final model retained the algorithm that maximized the positive‐predictive value (PPV) and the F1 score (the harmonic mean of the PPV and sensitivity). The robustness of the model was controlled using the crossed‐validation method, by dividing the total population studied into a training sample (70% of the individuals) and a validation sample (the remaining 30% of the subjects) with adjustment for age and sex.

Statistical analyses were coded in Python 3.7 and R with an R studio interface (version 3.6.2).

Authorization number 2210413v0 was accorded to access DRG data. The study was registered (number 4117120520) in the public directory of the Institut National de Données de Santé (INDS). The Ethics Committee of the Société de Pneumologie de Langue Française favorably approved the study (CEPRO 2020–021 of April 8, 2020). In accordance with French law, informing patients is not required for analyses conducted on anonymized data extracted from the DRG.

## 3 RESULTS

**FIGURE 1:**
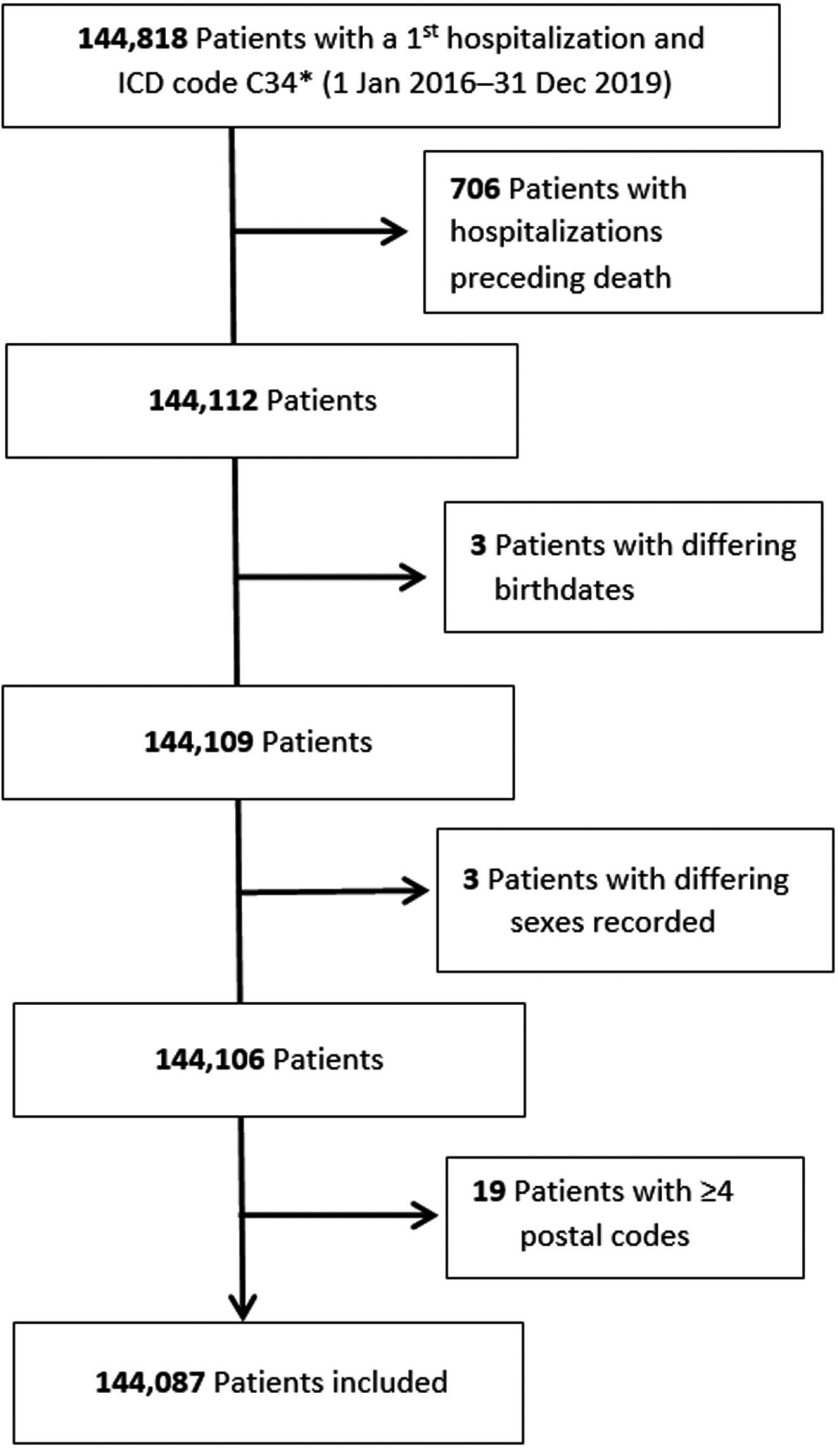
Flow chart

Among the 144,818 patients with a first hospitalization for lung cancer during the inclusion period, 144,087 (99.5%) were included for the analysis (Figure 1). They were predominantly men (67%); their median age was 68 years, with 17.2% >80 years old (Table 1); 34% were active smokers; 27%, 5%, 46%, or 18%, respectively, had the chronic obstructive pulmonary disease (COPD), chronic respiratory insufficiency, hypertension or diabetes; 47% had metastatic disease at diagnosis; 34% had received at least one systemic treatment during their management, and 23% underwent curative surgery. More than half were managed in general hospitals (40%) or university hospitals (26%) (Table 2); more than half resided in an urban zone; 36% had very poor access to care. According to the SDI, residence locations were balanced among the 4 classes. For a quarter of the patients, the first hospitalization for lung cancer occurred after consulting at the emergency department, and 5% of the patients were discharged from their first hospitalization to a palliative care unit. The median duration of the first hospital stay was 6 [1–13] days.

**TABLE 1:** Characteristics of the analyzed non‐selected lung‐cancer population

Characteristic	Total *n* = 144,087	Died <3 months *n* = 26,789	Died ≥3 months *n* = 117,298	*p* value
Age, years	68 [60–76]	70 [63–80]	67 [60–75]	<0.001
Male sex	96,068 (67)	19,442 (73)	76,626 (65)	<0.001
Smokers	48,908 (34)	7899 (29)	41,009 (35)	<0.001
Metastatic disease at diagnosis	67,787 (47)	20,270 (76)	47,517 (41)	<0.001
Systemic therapy	48,684 (34)	4261 (16)	44,423 (38)	<0.001
Curative surgery	33,377 (23)	844 (3)	32,533 (28)	<0.001
Comorbidities				
Chronic obstructive pulmonary disease	38,611 (27)	6511 (24)	32,100 (27)	<0.001
Chronic respiratory insufficiency	7170 (5)	1933 (7)	5237 (4)	<0.001
Hypertension	66,389 (46)	12,350 (46)	54,039 (46)	0.9318
Diabetes	26,447 (18)	5213 (19)	21,234 (18)	<0.001
Chronic renal insufficiency	280 (0.2)	90 (0.3)	190 (0.2)	<0.001
Palliative care consultation	53,541 (37)	14,036 (52)	39,505 (34)	<0.001
Days of 1st hospitalization	6 [1–13]	10 [4–19]	5 [1–11]	<0.001
Number of hospitalizations preceding year	0 [0–0]	0 [0–6]	0 [0–3]	<0.001
Number of ED consultations preceding year	0 [0–0]	0 [0–0]	0 [0–0]	<0.001

*Notes*: Results are expressed as *n* (%) or median [interquartile range].

Abbreviation: ED, emergency department.

**TABLE 2:** Medical‐administrative characteristics of the population analyzed

Characteristic	Total *n* = 144,087	Died <3 months *n* = 26,789	Died ≥3 months *n* = 117,298	*p*‐value
1st hospitalization from the ED	36,746 (26)	12,475 (47)	24,271 (21)	<0.001
Discharged from 1st hospitalization to LTCF	6763 (5)	735 (3)	6028 (5)	<0.001
Type of facility				<0.001
University hospital	36,911 (26)	5648 (21)	31,263 (27)	
General hospital	57,345 (40)	13,738 (51)	43,607 (37)	
Dedicated cancer center	7366 (5/6)	659 (2)	6707 (6)	
Other public	7447 (5/7)	1186 (4)	6261 (5)	
Private	33,858 (23/30)	5385 (21/20)	28,473 (24)	
Type of facility	1161 (1)	174 (1/0.6)	987 (1/0.8)	
Residential district				<0.001
Rural	48,479 (34/42)	9366 (35)	39,113 (33)	
Urban	84,194 (58/74)	15,485 (58)	68,709 (59)	
Unknown	11,414 (8/10)	1938 (7)	9476 (8)	
Population density				0.002
Sparse	39,902 (28/35)	7357 (27)	32,545 (28)	
Low	33,356 (23/29)	6132 (23)	27,224 (23)	
Intermediate/moderate	45,319 (31/40)	8581 (32)	36,738 (31)	
High	14,096 (10/12)	2781 (10)	11,315 (10)	
Unknown	11,414 (8)	1938 (7)	9476 (8)	
Access to care‐facility indicator				0.3
Very low	51,275 (36/45)	9633 (36)	41,642 (36)	
Weak	27,429 (19/24)	5139 (19)	22,290 (19)	
High	29,005 (20/25)	5312 (20)	23,693 (20)	
Very high	32,321 (22/28)	5967 (22)	26,354 (22)	
Unknown	4057 (3/4)	738 (3)	3319 (3)	
Social Deprivation Index				<0.001
Most underprivileged	28,143 (24/25)	5796 (22)	33,939 (24/29)	
Underprivileged	34,533 (24/30)	6372 (24)	28,161 (24)	
Privileged	39,320 (27/34)	7457 (28)	31,863 (27)	
Most privileged	33,682 (23/30)	6745 (25)	26,937 (23)	
Unknown (%)	2613 (2)	419 (2)	2194 (2)	

*Notes*: Results are expressed as *n* (%).

Abbreviations: ED, emergency department; LTCF, long‐term care facility.

Mortality at 3 months was 19% (stable over the 3 years analyzed). The patients dying early were significantly (*p* < 0.001): older, more frequently men, most often suffered from chronic respiratory insufficiency, more frequently had metastatic disease at diagnosis, less frequently underwent curative surgery or received systemic treatment, were most often hospitalized after consulting at the emergency department, and their first hospitalizations were significantly longer, after which they were significantly more frequently discharged to a long‐term care facility (LTCF).

Results of univariate and multivariate analyses are reported in Table 3. Multivariate analyses retained the following factors as being significantly and independently associated with early death: age, with a class effect for patients 50–59 years old and those >80 years old, compared to the 18–49‐year‐old class; male sex, metastatic disease at diagnosis, and first hospitalization after consulting at the emergency department.

**TABLE 3:** Uni‐ and multivariate analyses of factors associated with early mortality (≤3 months)

Factor	Univariate		Multivariate	
	OR	95% CI	Adjusted OR	95% CI
Age group, years				
18–49	1		1	
50–59	1.27	1.17–1.37	1.17	1.07–1.28
60–69	1.52	1.41–1.63	1.35	1.24–1.47
70–79	1.76	1.63–1.89	1.33	1.22–1.45
≥80	3.09	2.87–3.33	1.36	1.24–1.49
Male sex	1.40	1.36–1.45	1.50	1.44–1.55
Smoker	0.78	0.76–0.80	0.93	0.90–0.97
Metastatic disease at diagnosis	4.57	4.43–4.71	3.30	3.18–3.43
Specific treatment				
Systemic	0.31	0.30–0.32	0.22	0.21–0.23
Curative surgery	0.08	0.30–0.32	0.17	0.15–0.18
Comorbidities				
Chronic obstructive pulmonary disease	0.85	0.83–0.88	0.95	0.93–0.99
Chronic respiratory insufficiency	1.66	1.58–1.76	—	
Hypertension	1.00	0.97–1.03	—	
Diabetes	1.09	1.06–1.13	0.93	0.90–0.97
Chronic renal insufficiency	2.08	1.61–2.66	—	
Palliative care consultation	2.17	2.11–2.23	1.18	1.14–1.22
1st hospitalization from the ED	3.34	3.25–3.43	1.65	1.59–1.71
Discharged after 1st hospitalization to LTCF	0.52	0.48–0.56	—	
Duration of 1st hospitalization, days				
≤7	1		1	
8–14	1.93	1.86–1.99	1.40	1.34–1.46
15–30	3.26	3.15–3.38	1.64	1.57–1.71
31–90	3.48	3.30–3.66	1.58	1.49–1.68
Days of hospitalizations preceding year				
0	1		1	
1–6	1.40	1.34–1.47	1.23	1.16–1.29
≥7	1.85	1.78–1.93	1.42	1.35–1.5
Number of ED consultations preceding year	1.02	1.02–1.03	1.00	1.00–1.00
Type of facility				
University hospital	1		1	
General hospital	1.74	1.68–1.8	1.09	1.05–1.14
Dedicated cancer center	0.54	0.5–0.59	0.70	0.64–0.77
Other public	1.05	0.98–1.12	1.03	0.94–1.12
Private	1.05	1.01–1.09	1.09	1.04–1.15
Residential district				
Rural	1.06	1.03–1.09	1.05	1.00–1.11
Urban	1		1	
Population density				
Sparse	0.92	0.88–0.97	1.03	0.95–1.12
Low	0.92	0.87–0.96	1.00	0.93–1.08
Intermediate/moderate	0.95	0.91–1.00	0.99	0.94–1.05
High	1		1	
Access to care‐facility indicator				
Very low	1.02	0.99–1.06	—	
Weak	1.02	0.98–1.06	—	
High	0.99	0.95–1.03	—	
Very high	1		—	
Social deprivation index				
Most underprivileged	0.82	0.79–0.86	0.96	0.91–1.01
Underprivileged	0.90	0.87–0.94	1.00	0.95–1.05
Privileged	0.93	0.90–0.97	1.00	0.97–1.05
Most privileged	1		1	

Abbreviations: 95% CI: 95% confidence interval; ED, emergency department; LTCF, long‐term care facility; OR, odds ratio.

Systemically treated patients were significantly (*p* < 0.001) younger (64 [58–71] vs. 70 [62–79] years), had fewer comorbidities, were more frequently managed in dedicated cancer centers (9% vs. 3%) and were more rarely hospitalized after consulting at the emergency department (22% vs. 27%, *p* < 0.001). In contrast, no difference was found between the socioeconomic characteristics of patients who had or did not receive systemic treatment. The median interval between the first hospitalization and the first systemic treatment was 30 [0–79] days. The early‐mortality rate for the subgroup of systemically treated patients was 9%, significantly lower than for the patients who had not received a systemic treatment (24%, *p* < 0.001). However, the risk factors of early death for that subgroup were the same as for the general population (Table S1). Patients dying early were significantly (*p* < 0.001) older (66 [59–72] vs. 64 [58–71] years), and more frequently men (72% vs. 66%, *p* < 0.001), more often suffered from chronic respiratory insufficiency (5% vs. 3%), were more frequently hospitalized for the first time after consulting at the emergency department (34% vs. 21%), and their first hospitalizations were longer (10 [3–21] vs. 4 [1–11] days), after which they were discharged to an LTCF (2% vs. 4%). Their median interval between the first hospitalization and administration of systemic treatment was significantly shorter (0 [0–19] vs. 32 [0–84 days], *p* < 0.001).

Surgical patients, compared to those not undergoing surgery, were significantly (*p* < 0.001) younger (66 [59–72] vs. 68 [61–78] years), more often women (36% vs. 33%), and less frequently hospitalized for the first time after consulting at the emergency department (5% vs. 32%). The median interval between the first hospitalization and surgery was 36 [0–44] days. The early‐mortality rate for this subgroup of patients undergoing curative surgery was 3% (vs. 23% of those not having surgery, *p* < 0.001). The surgical patients that died early, compared to survivors, were significantly (*p* < 0.001) (Table S2) older (70 [64–77] vs. 66 [59–72] years), more frequently men (85% vs. 64%), had more frequently associated comorbidities (COPD: 40% vs. 33%; chronic respiratory insufficiency: 9% vs. 3%; hypertension: 57% vs. 49%; and diabetes: 22% vs. 7%), had more often been hospitalized after consulting at the emergency department (15% vs. 4%) and their first hospitalizations lasted longer (8 [3–15] vs. 6 [1–9] days), after which they were more often discharged to an LTCF (3% vs. 6%). They more frequently lived in a zone with a not very dense population (27% vs. 29% *p* = 0.04). Their other socioeconomic characteristics did not differ.

After excluding patients with missing information, data from 129,788 patients were used for modelization. Because its collection was not reliable, the variable for tobacco use was excluded. The best performing logistic‐regression model (Table 4) had 46% sensitivity, 88% specificity, and an F1 score of 0.44. Having metastatic‐at‐diagnosis lung cancer and the first hospitalization from the emergency department were the two most discriminating variables (Table 5). Applying the model to the validation cohort showed performance stability. When only non‐surgical patients were included in the model (99,883 patients and 23% early deaths), performance indexes were slightly modified, with respective F1 scores and PPV of 0.44 and 42%. For this non‐surgical population, the metastatic disease status was less discriminating than for the general lung‐cancer population but nonetheless remained the most important.

**TABLE 4:** Performances of the predictive models for early mortality (≤3 months)

Parameter	Logistic regression	Decision tree	SVM
F1 score	0.44	0.39	0.46
Positive‐predictive value	41%	038%	33%
Sensitivity	46%	040%	77%
Accuracy	77%	76%	65%
Specificity	88%	85%	63%

Abbreviation: SVM, support vector machine (i.e., supervised learning model).

**TABLE 5:** Model variables and coefficients

Variable	Coefficient
Metastatic disease at diagnosis	1.319
1st hospitalization from the ED	0.740
Duration of hospitalization preceding year ≥7 days	0.421
Age normalized	0.304
Managed in a hospital	0.207
Managed in a private facility	0.207
Chronic renal insufficiency	0.163
Duration of 1st hospitalization (standardized)	0.140
Duration of hospitalization preceding year 1–6 days	0.097
Social deprivation index	0.054
Number of ED consultations the preceding year	0.007
Management in a university hospital	0.004
Living in a very low‐dense zone	−0.033
Diabetes	−0.037
Living in an urban zone	−0.056
Living in a moderate‐density zone	−0.061
Living in a low‐density zone	−0.074
Chronic obstructive pulmonary disease	−0.084
Hypertension	−0.230
Female	−0.391
Managed in a dedicated cancer center	−0.512

Abbreviation: ED, emergency department.

The contribution of this model predictive of early mortality to clinical practice is illustrated by the following two hypothetical cases. First, the probability of dying within the 3 months following the first hospitalization for a 68‐year‐old woman—diagnosed with localized lung cancer, not hospitalized after consulting at the emergency department, with hypertension and COPD, managed in a private facility, with a 1‐day first hospitalization, 14 cumulative days of hospitalization the preceding year and residing in an urban zone of intermediate density and privileged SDI—is 6%. Second, the probability of early death for a 78‐year‐old man—hospitalized from the emergency department, diagnosed with metastatic lung cancer, hypertension, COPD, and diabetes, managed in a general hospital with a first hospitalization lasting 82 days, 39 cumulative days of hospitalization during the preceding year, living in a population‐dense, urban zone, and privileged SDI–is 77%.

## 4 DISCUSSION

The results of this study show that, in France, early mortality by 3 months of patients newly diagnosed with lung cancer was 19%, and that rate increased significantly with age, for patients with metastatic disease at diagnosis, for men, and when the first hospitalization admission was from the emergency department.

This early‐mortality rate of 19% was lower than that found in other studies. In their 2010 prospective, multicenter study that included 7051 patients with newly diagnosed lung cancers, Grivaux et al.^15^ reported 23.2% 3‐month mortality. According to an analysis of a United Kingdom medical‐administrative database with 20,142 patients diagnosed with lung cancer between January 2000 and January 2013, 3‐month mortality was 30.2%.^14^ These differences can be explained, in part, by the methodologies used. Grivaux et al.^3^ included only patients managed in general hospitals, older and more often with metastatic disease at diagnosis than those diagnosed at the national level.^6^ The British study^14^ also included patients entered in a database of general medicine consultations and, thus, excluded other diagnosis sources.

Certain factors associated with early mortality highlighted in our analysis were previously reported, especially age,^13^, ^15^, ^18^, ^26^ metastatic disease at diagnosis,^8^, ^15^ and male sex.^13^, ^14^, ^15^, ^18^ A recent study of the SEER registry about gender and lung cancer^27^ suggests found that male patients were more likely than female patients to be diagnosed at stage III or IV, consistent across lung cancer types, cancer registries, smoking, and socioeconomic backgrounds. In our population, women are younger than men (67 vs. 68 years) and have more often surgery (24.7 vs. 22.4%). These characteristics may explain why their survival rate is better but it is to note that there are also more often diagnosed at a metastatic stage (32.4 vs. 30.8%). Other factors that are not available in our database, such as histologic cancer type or performance status, may also explain the difference.

In contrast, characteristics associated with the first hospitalization for lung cancer (pathway of entry and duration of stay) have rarely been examined and the findings were contradictory.

According to a single‐center, retrospective analysis of 771 advanced NSCLC patients,^28^ 103 (13%) were diagnosed with lung cancer after consulting at the emergency department. Their multivariate analysis did not retain diagnosis after consulting at the emergency department as having an impact on global mortality, but early mortality was not reported. On the contrary, an analysis of 133,530 NSCLC patients from the UK National Lung Cancer Audit revealed that 19% of them were hospitalized after consulting at the emergency department. This care pathway was strongly associated with more advanced disease stages, poorer performance status, more unfavorable SDI, and very old age.^29^ The probability of having died within 1‐year post‐diagnosis was higher after adjusting for important clinical parameters.

Findings were also contradictory about the impact of socioeconomic characteristics on early mortality. We did not find significant associations between early mortality and population density or SDI status of the patient's zone of residence. Higher early mortality in rural zones was identified in certain studies,^14^ whereas early mortality for the same zones was significantly lower in others.^30^ These contradictions probably reflect the different organizations of healthcare systems and differing definitions of rurality. In addition, residing in an underprivileged zone is often associated with significantly higher rates of early lung cancer deaths,^14^, ^18^, ^26^ but those investigations used different criteria than we did to define socially deprived status.

Little has been published on the impact of comorbidities on early mortality from lung cancer. Concerning global mortality, concomitant pulmonary diseases (chronic bronchopathy, pulmonary fibrosis, tuberculosis) increased the risk of death.^31^ Moreover, according to a population‐based study on 5683 patients with newly diagnosed lung cancer, various comorbidities were negatively associated with adjusted overall survival.^32^ In that study population, 26.7% of the subjects had no comorbidity at lung cancer diagnosis. In our study population, 55,402 (38.5%) patients had no comorbidities but having comorbidities seemed to have a low impact on early mortality.

Several teams have tried to develop models predictive of early mortality, either for locally advanced lung cancers^11^, ^33^ or small‐cell lung cancers.^34^ Those models were constructed with smaller samples of patients and integrated only clinical and laboratory criteria. The predictive model devised herein, by integrating information associated with the characteristics of the first hospitalization, the disease‐managing facility, and the characteristics of the patient's area of residence, performed modestly but could nevertheless contribute to lung cancer management by identifying patients at high risk of early death in a non‐selected population.

One of the strengths of the study is the national and exhaustive character of the database including all healthcare structures, private and public, palliative care units, and day hospitals. But this study has also several limitations. The analysis was based on a medical‐administrative database that did not contain certain factors that could have had an impact on early mortality, especially tumor histology, race, marital status, T and N stage, bone and liver metastasis, and performance status. Other variables were only imperfectly coded, e.g., smoking history, which in this analysis, and surprisingly, had a protective role against early death, whereas it had been reported to be a risk factor for death at 3 months, with ORs of 1.43 (1.28–1.61)^14^ and 1.36 (1.03–1.81).^3^ This discordance probably resulted from faulty coding and loss of information on the active status or not. Indeed in our population, only 34% are identified as smokers while in other French studies,^3^, ^16^ smokers, or former smokers represent 88% of patients. Another limitation is that the SDI variables and access to care‐facility indicator were established at the district level and not individually. Some patients may not have been taken into account, in particular those who were diagnosed with lung cancer in an outpatient setting and who died before any hospitalization. Finally, the modeling used hypothetical cases which do not necessarily reflect the average of the cases in the cohort

## 5 CONCLUSION

At the national level, almost 1 in 5 patients diagnosed with lung cancer died within 3 months following the first hospitalization. Early mortality is higher for patients ≥70 years, for men, for those with metastatic disease at diagnosis, for patients hospitalized via the emergency room, and with first hospitalization lasting >30 days. No socioeconomic characteristic is associated with early mortality. Improving survival requires diagnosis at an earlier stage and better organization of diagnosis and specific care pathways.

## CONFLICT OF INTEREST

The authors have no conflicts of interest to declare. This research did not receive any specific grant from funding agencies in the public, commercial, or not‐for‐profit sectors.

## AUTHOR CONTRIBUTIONS

Conception and design: H. Goussault, C. Chouaid. Administrative support: C. Jung, M. Rousseau, A. Lazatti. Provision of study materials or patients: S. Epaud, A. Lazatti. Collection and assembly of data: H. Goussault, S. Epaud, N. Salaun‐Penquer, C. Algans. Data analysis and interpretation: H. Goussault, C. Algans. Manuscript writing: All authors. Final approval of manuscript: All authors.

## ETHICAL STATEMENT

The authors are accountable for all aspects of the work in ensuring that questions related to the accuracy or integrity of any part of the work are appropriately investigated and resolved. The study was approved by the Ethics Committee of the (Société de Pneumologie de Langue Française on April 8, 2020).

## Supporting information


Appendix

## Data Availability

The data that support the findings of this study are available from the corresponding author, HG.

## References

[1] The Global Cancer Observatory. https://gco.iarc.fr/today/home

[2] Howlader N , Forjaz G , Mooradian MJ , et al. The effect of advances in lung‐cancer treatment on population mortality. N Engl J Med. 2020;383(7):640‐649. 32786189 10.1056/NEJMoa1916623 PMC8577315

[3] Colonna M . Epidémiologie du cancer du poumon en France: incidence, mortalité et survie (tendance et situation actuelle). Rev mal Respir Actual. 2016;8(5):308‐318.

[4] Recommandations professionnelles Cancer du poumon non à petites cellules Formes localisées non opérables, localement avancées et métastatiques. Collection Recommandations & référentiels, INCa; 2010: p. 5.

[5] Kawaguchi T , Takada M , Kubo A , et al. Performance status and smoking status are independent favorable prognostic factors for survival in non‐small cell lung cancer: a comprehensive analysis of 26,957 patients with NSCLC. J Thorac Oncol. 2010;5(5):620‐630. 20354456 10.1097/JTO.0b013e3181d2dcd9

[6] Chouaïd C , Debieuvre D , Durand‐Zaleski I , et al. Survival inequalities in patients with lung cancer in France: a nationwide cohort study (the TERRITOIRE Study). PLOS ONE. 2017;12(8):e0182798. 28841679 10.1371/journal.pone.0182798 PMC5571949

[7] Finke I , Behrens G , Weisser L , Brenner H , Jansen L . Socioeconomic differences and lung cancer survival—systematic review and meta‐analysis. Front Oncol. 2018;8:536. https://www.frontiersin.org/article/10.3389/fonc.2018.00536/full 30542641 10.3389/fonc.2018.00536 PMC6277796

[8] Melvan JN , Sancheti MS , Gillespie T , et al. Nonclinical factors associated with 30‐day mortality after lung cancer resection: an analysis of 215,000 patients using the National Cancer Data Base. J Am Coll Surg. 2015;221(2):550‐563. 26206651 10.1016/j.jamcollsurg.2015.03.056 PMC4514912

[9] Thomas P , Piraux M , Jacques LF , Grégoire J , Bédard P , Deslauriers J . Clinical patterns and trends of outcome of elderly patients with bronchogenic carcinoma. Eur J Cardio‐Thorac Surg off J Eur Assoc Cardio‐Thorac Surg. 1998;13(3):266‐274. 10.1016/s1010-7940(98)00011-6 9628376

[10] Ludwig C , Stoelben E , Olschewski M , Hasse J . Comparison of morbidity, 30‐day mortality, and long‐term survival after pneumonectomy and sleeve lobectomy for non‐small cell lung carcinoma. Ann Thorac Surg. 2005;79(3):968‐973. 15734415 10.1016/j.athoracsur.2004.08.062

[11] Klement RJ , Belderbos J , Grills I , et al. Prediction of early death in patients with early‐stage NSCLC‐can we select patients without a potential benefit of SBRT as a curative treatment approach? J Thorac Oncol off Publ Int Assoc Study Lung Cancer. 2016;11(7):1132‐1139. 10.1016/j.jtho.2016.03.016 27060654

[12] Baker S , Sharma A , Peric R , Heemsbergen WD , Nuyttens JJ . Prediction of early mortality following stereotactic body radiotherapy for peripheral early‐stage lung cancer. Acta Oncol Stockh Swed. 2019;58(2):237‐242. 10.1080/0284186X.2018.1532602 30451552

[13] Morgensztern D , Samson PS , Waqar SN , et al. Early mortality in patients undergoing adjuvant chemotherapy for non‐small cell lung cancer. J Thorac Oncol off Publ Int Assoc Study Lung Cancer. 2018;13(4):543‐549. 10.1016/j.jtho.2018.01.010 29410127

[14] O'Dowd EL , McKeever TM , Baldwin DR , et al. What characteristics of primary care and patients are associated with early death in patients with lung cancer in the UK? Thorax. 2015;70(2):161‐168. 25311471 10.1136/thoraxjnl-2014-205692 PMC4316923

[15] Grivaux M , Debieuvre D , Herman D , et al. Early mortality in lung cancer: French prospective multicentre observational study. BMC Pulm Med. 2016;16:45. 27039176 10.1186/s12890-016-0205-5 PMC4818853

[16] Quoix E , Monnet I , Scheid P , et al. l'Intergroupe francophone de cancérologie thoracique (IFCT)Management and outcome of French elderly patients with lung cancer: an IFCT survey. Rev mal Respir. 2010;27(5):421‐430. 20569874 10.1016/j.rmr.2010.02.013

[17] Winter MC , Potter VA , Woll PJ . Raised serum urea predicts for early death in small cell lung cancer. Clin Oncol R Coll Radiol. 2008;20(10):745‐750. 18845424 10.1016/j.clon.2008.09.001

[18] Powell HA , Tata LJ , Baldwin DR , Stanley RA , Khakwani A , Hubbard RB . Early mortality after surgical resection for lung cancer: an analysis of the English National Lung cancer audit. Thorax. 2013;68(9):826‐834. 23687050 10.1136/thoraxjnl-2012-203123

[19] Stoelben E , Sauerbrei W , Ludwig C , Hasse J . Tumor stage and early mortality for surgical resections in lung cancer. Langenbecks Arch Surg. 2003;388(2):116‐121. 12712342 10.1007/s00423-003-0354-x

[20] Bezin J , Duong M , Lassalle R , et al. The national healthcare system claims databases in France, SNIIRAM and EGB: powerful tools for pharmacoepidemiology. Pharmacoepidemiol Drug Saf. 2017;26(8):954‐962. 28544284 10.1002/pds.4233

[21] Correspondance Code INSEE ‐ Code Postal 2013. Disponible sur: https://public.opendatasoft.com/explore/dataset/correspondance‐code‐insee‐code‐postal/information/?dataChart=eyJxdWVyaWVzIjpbeyJjb25maWciOnsiZGF0YXNldCI6ImNvcnJlc3BvbmRhbmNlLWNvZGUtaW5zZWUtY29kZS1wb3N0YWwiLCJvcHRpb25zIjp7fX0sImNoYXJ0cyI6W3siYWxpZ25Nb250aCI6dHJ1ZSwidHlwZSI6ImNvbHVtbiIsImZ1bmMiOiJBVkciLCJ5QXhpcyI6InpfbW95ZW4iLCJzY2llbnRpZmljRGlzcGxheSI6dHJ1ZSwiY29sb3IiOiIjRkY1MTVBIn1dLCJ4QXhpcyI6Imluc2VlX2NvbSIsIm1heHBvaW50cyI6NTAsInNvcnQiOiIifV0sInRpbWVzY2FsZSI6IiIsImRpc3BsYXlMZWdlbmQiOnRydWUsImFsaWduTW9udGgiOnRydWV9

[22] INSEE. La grille communale de densité. https://www.insee.fr/fr/information/2114627

[23] L'indicateur d'accessibilité potentielle localisée (APL) aux principaux professionnels de soins de premier recours. Disponible sur: http://www.data.drees.sante.gouv.fr/TableViewer/document.aspx?ReportId=4475

[24] Rey G , Jougla E , Fouillet A , Hémon D . Ecological association between a deprivation index and mortality in France over the period 1997–2001: variations with spatial scale, degree of urbanicity, age, gender and cause of death. BMC Public Health. 2009;9(1):33. 19161613 10.1186/1471-2458-9-33 PMC2637240

[25] Ministère des Solidarités et de la Santé. FINESS Extraction du Fichier des établissements. Disponible sur: https://www.data.gouv.fr/fr/datasets/finess‐extraction‐du‐fichier‐des‐etablissements/

[26] McPhail S , Johnson S , Greenberg D , Peake M , Rous B . Stage at diagnosis and early mortality from cancer in England. Br J Cancer. 2015;112(S1):S108‐S115. 25734389 10.1038/bjc.2015.49 PMC4385983

[27] Tolwin Y , Gillis R , Peled N . Gender and lung cancer—SEER‐based analysis. Ann Epidemiol. 2020;46:14‐19. 32532368 10.1016/j.annepidem.2020.04.003

[28] Fujimoto D , Shimizu R , Morimoto T , et al. Analysis of advanced lung cancer patients diagnosed following emergency admission. Eur Respir J. 2015;45(4):1098‐1107. 25323241 10.1183/09031936.00068114

[29] Beckett P , Tata LJ , Hubbard RB . Risk factors and survival outcome for non‐elective referral in non‐small cell lung cancer patients – analysis based on the National Lung Cancer Audit. Lung Cancer. 2014;83(3):396‐400. 24457105 10.1016/j.lungcan.2013.10.010

[30] Amini A , Verma V , Glaser SM , et al. Early mortality of stage IV non‐small cell lung cancer in the United States. Acta Oncol. 2019;58(8):1095‐1101. 30958075 10.1080/0284186X.2019.1599138

[31] Gao Y , Guan W , Liu Q , et al. Impact of COPD and emphysema on survival of patients with lung cancer: a meta‐analysis of observational studies. Respirology. 2016;21(2):269‐279. 26567533 10.1111/resp.12661

[32] Islam KMM , Jiang X , Anggondowati T , Lin G , Ganti AK . Comorbidity and survival in lung cancer patients. Cancer Epidemiol Biomarkers Prev. 2015;24(7):1079‐1085. 26065838 10.1158/1055-9965.EPI-15-0036

[33] Jochems A , El‐Naqa I , Kessler M , et al. A prediction model for early death in non‐small cell lung cancer patients following curative‐intent chemoradiotherapy. Acta Oncol Stockh Swed. 2018;57(2):226‐230. 10.1080/0284186X.2017.1385842 PMC6108087 29034756

[34] Quoix E , Hedelin G , Popin E , et al. Can we predict very short term survival in small cell lung cancer? Lung Cancer. 1993;10(3–4):229‐238. 8075968 10.1016/0169-5002(93)90183-x

