# Supplementary material for: Risk factors for early mortality of lung cancer patients in France: A nationwide analysis
Source: Cancer Med. 2022 May 14;11(24):5025–34. doi: 10.1002/cam4.4821 (PMC9761075; doi:10.1002/cam4.4821)
Supplement: Supplementary file 1 — Appendix [file CAM4-11-5025-s001.docx]

**Appendices**

**Table S1** Uni- and multivariate analyses of factors associated with early mortality (≤3 months) for the subgroup receiving systemic therapy

| **Factor** | **Univariate** | |  | **Multivariate** | | |
| --- | --- | --- | --- | --- | --- | --- |
|  | **OR** | **95% CI** |  | **Adjusted OR** | | **95% CI** |
| Age group, years |  |  |  |  |  | |
| 18–49 | 1 |  |  | 1 |  | |
| 50–59 | 1.08 | 0.94–1.25 |  | 1.09 | 0.94–1.26 | |
| 60–69 | 1.26 | 1.11–1.45 |  | 1.28 | 1.11–1.47 | |
| 70–79 | 1.48 | 1.29–1.70 |  | 1.47 | 1.28–1.7 | |
| ≥80 | 1.63 | 1.38–1.93 |  | 1.52 | 1.28–1.8 | |
| Male sex | 1.32 | 1.23–1.42 |  | 1.31 | 1.22–1.41 | |
| Comorbidities |  |  |  |  |  | |
| Chronic obstructive pulmonary disease | 0.76 | 0.71–0.82 |  | 0.71 | 0.66–0.77 | |
| Chronic respiratory insufficiency | 1.5 | 1.3–1.73 |  | – |  | |
| Hypertension | 0.95 | 0.9–1.02 |  | – |  | |
| Diabetes | 1.03 | 0.95–1.12 |  | – |  | |
| Chronic renal insufficiency | 1.96 | 0.9–3.8 |  | – |  | |
| 1^st^ hospitalization from the ED | 1.97 | 1.84–2.11 |  | 1.35 | 1.25–1.46 | |
| 1^st^ hospitalization discharged to LTCF | 0.57 | 0.46–0.69 |  | 0.31 | 0.25–0.37 | |
| Days of 1^st^ hospitalization |  |  |  |  |  | |
| ≤7 | 1 |  |  | 1 |  | |
| 8–14 | 1.73 | 1.59–1.89 |  | 1.66 | 1.52–1.82 | |
| 15–30 | 2.84 | 2.62–3.08 |  | 2.73 | 2.5–2.98 | |
| 31–90 | 3.79 | 3.42–4.2 |  | 3.93 | 3.52–4.39 | |
| Type of facility |  |  |  |  |  | |
| University hospital | 1 |  |  | 1 |  | |
| General Hospital | 1.34 | 1.24–1.46 |  | 1.23 | 1.13–1.34 | |
| Dedicated cancer center | 0.74 | 0.64–0.85 |  | 0.95 | 0.82–1.1 | |
| Other public | 0.91 | 0.76–1.08 |  | 0.97 | 0.81–1.16 | |
| Private | 1.44 | 1.31–1.58 |  | 1.6 | 1.45–1.76 | |
| Residential district |  |  |  |  |  | |
| Rural | 1.06 | 0.99–1.13 |  | – |  | |
| Urban | 1 |  |  | – |  | |
| Population density |  |  |  |  |  | |
| Sparse | 0.95 | 0.85–1.07 |  | – |  | |
| Low | 0.98 | 0.87–1.1 |  | – |  | |
| Moderate | 1.03 | 0.92–1.15 |  | – |  | |
| High | 1 |  |  | – |  | |
| Access to care-facility indicator |  |  |  |  |  | |
| Very low | 0.98 | 0.9–1.07 |  | – |  | |
| Weak | 1 | 0.91–1.1 |  | – |  | |
| High | 0.98 | 0.89–1.08 |  | – |  | |
| Very high | 1 |  |  | – |  | |
| Social Deprivation Index |  |  |  |  |  | |
| Most underprivileged | 0.93 | 0.85–1.02 |  | – |  | |
| Underprivileged | 1.05 | 0.96–1.15 |  | – |  | |
| Privileged | 1.02 | 0.93–1.11 |  | – |  | |
| Most privileged | 1 |  |  | – |  | |

OR: odds ratio; 95% CI: 95% confidence interval; ED: emergency department; LTCF: long-term–care facility.

**Table S2** Uni- and multivariate analyses of factors associated with early mortality (≤3 months) for the subgroup undergoing curative surgery

| **Factor** | **Univariate** | |  | **Multivariate** | |
| --- | --- | --- | --- | --- | --- |
|  | **OR** | **95% CI** |  | **Adjusted OR** | **95% CI** |
| Age group, years |  |  |  |  |  |
| 18–49 | 1 |  |  | 1 |  |
| 50–59 | 1.53 | 0.94–2.63 |  | 1.44 | 0.88–2.49 |
| 60–69 | 2.56 | 1.64–4.29 |  | 2.32 | 1.47–3.91 |
| 70–79 | 3.98 | 2.54–6.65 |  | 3.5 | 2.21–5.91 |
| ≥80 | 8.96 | 5.62–15.2 |  | 7.42 | 4.59–12.74 |
| Male sex | 3.19 | 2.65–3.88 |  | 2.65 | 2.19–3.24 |
| Comorbidities |  |  |  |  |  |
| Chronic obstructive pulmonary disease | 1.39 | 1.21–1.6 |  | 1.07 | 0.92–1.24 |
| Chronic respiratory insufficiency | 3.23 | 2.53–4.08 |  | – |  |
| Hypertension | 1.41 | 1.23–1.62 |  | 0.95 | 0.82–1.11 |
| Diabetes | 1.37 | 1.16–1.62 |  | 1.03 | 0.86–1.22 |
| Chronic renal insufficiency | 3.97 | 1.19–9.88 |  | – |  |
| 1^st^ hospitalization from ED | 3.8 | 3.11–4.61 |  | 2.52 | 2.02–3.13 |
| Discharged from 1^st^ hospitalization to LTCF | 0.57 | 0.38–0.81 |  | 0.37 | 0.24–0.53 |
| Days of 1^st^ hospitalization |  |  |  |  |  |
| ≤7 | 1 |  |  | 1 |  |
| 8–14 | 1.15 | 0.97–1.37 |  | 1.12 | 0.94–1.33 |
| 15–30 | 3.2 | 2.65–3.86 |  | 2.74 | 2.24–3.34 |
| 31–90 | 9.45 | 7.23–12.21 |  | 8.2 | 6.17–10.79 |
| Type of facility |  |  |  |  |  |
| University hospital | 1 |  |  | 1 |  |
| General hospital | 1.22 | 1.02–1.45 |  | 0.97 | 0.81–1.17 |
| Dedicated cancer center | 0.51 | 0.31–0.79 |  | 0.68 | 0.41–1.06 |
| Other public | 0.93 | 0.68–1.23 |  | 0.92 | 0.67–1.22 |
| Private | 0.99 | 0.83–1.18 |  | 1.05 | 0.88–1.25 |
| Residential district |  |  |  |  |  |
| Rural | 1.07 | 0.92–1.24 |  | – |  |
| Urban | 1 |  |  | – |  |
| Population density |  |  |  |  |  |
| Sparse | 1.03 | 0.79–1.36 |  | – |  |
| Low | 1.07 | 0.82–1.42 |  | – |  |
| Moderate | 1.29 | 1–1.68 |  | – |  |
| High | 1 |  |  | – |  |
| Access to care-facility indicator |  |  |  |  |  |
| Very low | 0.89 | 0.75–1.07 |  | – |  |
| Weak | 0.84 | 0.68–1.04 |  | – |  |
| High | 0.92 | 0.75–1.13 |  | – |  |
| Very high | 1 |  |  | – |  |
| Social Deprivation Index |  |  |  |  |  |
| Most underprivileged | 0.88 | 0.72–1.07 |  | – |  |
| Underprivileged | 0.94 | 0.77–1.15 |  | – |  |
| Privileged | 1.07 | 0.88–1.3 |  | – |  |
| Most privileged | 1 |  |  | – |  |

OR: odds ratio; 95% CI: confidence interval; ED, emergency department; LTCF: long-term–care facility.
